# The grapevine LysM receptor kinase VvLYK4-2 is a key player in chitosan-triggered immune responses

**DOI:** 10.1093/hr/uhag097

**Published:** 2026-03-13

**Authors:** Thibault Roudaire, Jérémy Villette, Tania Marzari, Daphnée Brulé, Stéphanie Pradeau, Sébastien Fort, David Landry, Benoit Lefebvre, Marie-Claire Héloir, Benoit Poinssot

**Affiliations:** Université Bourgogne Europe, INRAE, Institut Agro, UMR Agroécologie, Dijon, France; Université Bourgogne Europe, INRAE, Institut Agro, UMR Agroécologie, Dijon, France; Université Bourgogne Europe, INRAE, Institut Agro, UMR Agroécologie, Dijon, France; Université Bourgogne Europe, INRAE, Institut Agro, UMR Agroécologie, Dijon, France; CNRS, Univ. Grenoble Alpes, CERMAV, Grenoble, France; CNRS, Univ. Grenoble Alpes, CERMAV, Grenoble, France; Univ. Toulouse, CNRS, INRAE, LIPME, Castanet-Tolosan, France; Univ. Toulouse, CNRS, INRAE, LIPME, Castanet-Tolosan, France; Université Bourgogne Europe, INRAE, Institut Agro, UMR Agroécologie, Dijon, France; Université Bourgogne Europe, INRAE, Institut Agro, UMR Agroécologie, Dijon, France

## Abstract

Chitooligosaccharides, such as chitin, are essential components of fungal cell walls and have thus naturally been selected as microbe-associated molecular patterns detected by plants to initiate defense mechanisms. These molecules are typically recognized by the lysin motif receptor-like kinases (LysM RLKs) at the plasma membrane. While chitin perception is well elucidated in *Arabidopsis thaliana* and other plant species, the recognition mechanisms of its deacetylated form, chitosan, remain poorly investigated despite its use as a biocontrol strategy to protect crops against pathogens. Here, we investigated the role of the two grapevine orthologs of *AtLYK4*, which participate in the tripartite complex for chitin perception in *A. thaliana*. Using a dual approach consisting of the functional complementation of the *atlyk4/5* double mutant and CRISPR-Cas9 genome editing in *Vitis vinifera*, we showed that *VvLYK4-2* is involved in both chitosan- and chitin-induced immune responses, encompassing MAPK phosphorylation and defense gene expression. Furthermore, grapevine *in vitro* plantlets lacking *VvLYK4-2* exhibited a significantly reduced response to chitosan while retaining a low-intensity response to chitin, potentially due to the presence of *VvLYK5-1*. Finally, VvLYK4-2 produced in a heterologous system showed binding to chitosan oligomers and, to a lesser extent, to chitin oligomers. These findings indicate that this pattern recognition receptor plays a crucial role in the perception of chitosan oligomers and has thus potential for selective breeding purposes. This discovery may also help to better understand the partial lack of efficacy of chitosan-based plant defense stimulants used in viticulture.

## Introduction

Plants have a multitude of plasma membrane-bound receptors, known as pattern recognition receptors (PRR), which allow them to distinguish between different danger signals such as pathogen/microbe-associated molecular patterns (PAMP/MAMP) to mount appropriate responses regarding the situation they are facing [1]. This recognition generally involves PRRs with a strong affinity for the ligand, and PRRs that have a signaling role and act as co-receptors able to integrate and transduce many external signals [2]. As such, MAMP perception generally involves two or more partners forming immune receptor complexes at the plasma membrane. After complex formation, a molecular signal is transmitted inside the cell. Although proteins allowing this signal transduction can differ according to the immune complex involved in the MAMP perception, it generally involves the activation of cytosolic protein kinases (CPKs), including the well-described mitogen-activated protein kinase (MAPK) cascade. These kinases transmit the signal through phosphorylation and lead to the activation of transcription factors, ultimately leading to transcriptome reprogramming [3]. Signal transduction is also directed by ions flux across the plasma membrane, which occurs within seconds following ligand perception, and can often be accompanied by a later reactive oxygen species (ROS) production mediated by the activity of NADPH oxidases [4]. Transcriptome reprogramming leads to the activation of defense genes encoding, for example, pathogenesis-related (PR) proteins (i.e. chitinases or proteases) and phytoalexins with antimicrobial properties [2]. If these defenses are efficient enough, they can finally stop the pathogen spreading and conduct to the PAMP/MAMP triggered immunity (PTI/MTI).

PRRs are categorized into different groups according to the extracellular domain they harbor [2]. The majority of plant PRRs are leucine-rich repeat (LRR) receptors, which allow the recognition of a wide range of peptides, but plants also possess lysin-motif (LysM) receptors, allowing them to perceive several oligosaccharides [2, 5]. Among them, chitin, a polymer of N-acetyl-D-glucosamine (GlcNAc) composing the fungal cell walls and arthropod cuticles, has been widely studied because of its properties to elicit defenses in a large array of plant species including both mono- and eudicotyledons [6–9]. Chitin perception is well described in rice and in the model plant *Arabidopsis thaliana* where it mainly involves a LysM receptor-like kinase (LysM-RLK) with a functional kinase domain, named CERK1, and a LysM-PRR lacking this domain (OsCEBIP; a rice LysM domain-containing glycosylphosphatidylinositol-anchored protein) or having lost its autophosphorylation activity (AtLYK5; an *A. thaliana* LysM-RLK) [6, 7, 10, 11]. In the current *A. thaliana* chitin perception model, AtLYK5 is proposed to form heterodimeric complexes with AtLYK4 at the basal state [12, 13]. After chitin perception by AtLYK5, this receptor has been shown to directly interact with AtCERK1 to induce immune signaling [11]. Whereas analysis of *atcerk1* and *atlyk5* mutants revealed a strongly altered phenotype of chitin-induced immune responses, analysis of the *atlyk4* mutant only highlighted a weak decrease of these responses [7, 11, 14] (i.e. calcium influx, ROS production, defense gene expression, and callose deposition) and a variable sensitivity towards fungal or bacterial pathogens [11, 12, 15]. It is not clear if AtLYK4 could directly bind chitin oligomers, as conflicting experiments have both revealed chitin binding by heterologous production of the extracellular part (ECD) of AtLYK4 in bacteria [12] and no affinity for chitin hexamers after its production in insect cells, while a weak association has been detected with the AtCERK1-ECD [11, 16]. As no direct interaction could be shown between AtCERK1 and AtLYK4, it was proposed that AtLYK4 could act as a scaffold protein to stabilize AtCERK1-AtLYK5 complex by interacting with AtLYK5 and thus improving the chitin-induced immunity [12]. Furthermore, since AtLYK4 can interact with AtLYM2 and co-localizes with this LysM-RLP at the plasmodesmata, it was also suggested to play a role in AtLYM2-mediated chitin-induced plasmodesmal closure [13].

Although chitin is a well-known elicitor of plant defenses, it has not been widely adopted for plant protection purposes. However, chitosan, its deacetylated derivative, is an active ingredient of homologated biocontrol products in Europe (i.e. COS-OGA-based products; [17]) and allows good protection levels against oomycetes and fungal pathogens for different species, including grapevine and Arabidopsis [18, 19], thanks to its dual action as a plant defense elicitor and antimicrobial agent [19, 20]. Chitosan perception on both species is unfortunately poorly described, complicating the improvement of these bio-based plant protection products. In Arabidopsis, the mechanisms of chitosan perception remain unexplained. Some studies argue for an AtCERK1-independent perception of chitosan, since AtCERK1 is unable to bind this molecule [21] and chitosan can induce the expression of defense genes in both wild-type (WT) and in the Arabidopsis-deficient mutant *atcerk1* [18]. For others, chitosan could act in a CERK1- and PRR-independent manner by disturbing phospholipids at the plasma membrane [22]. However, this latter hypothesis does not explain the absence of MAPK activation and the reduced *FRK1* gene expression following a chitosan treatment in the *atcerk1* mutant line, highlighted by Brulé *et al*. [9], nor the refractory state observed after a second chitosan application related to MAMP perception by high-affinity receptors [22, 23]. Regarding the ability of two grapevine (*Vitis vinifera*) orthologs of *AtCERK1* (*VvLYK1-1* and *VvLYK1-2*) to restore MAPK activation and *FRK1* gene expression following a chitosan treatment in the *atcerk1* mutant line [9], it is likely that chitosan perception is mediated by these LysM-PRRs.

We recently showed that chitin perception in grapevine was allowed by both VvLYK5-1 and VvLYK1-1, which interact together after chitin treatment [9, 24]. Although we clearly established that chitosan hexamer was not perceived by the grapevine VvLYK5-1 and VvLYK5-2 PRRs, we highlighted strongly reduced chitosan-induced immune responses in the *atlyk4/5* double mutant [24]. In the present work, we investigated the role of *VvLYK4-1/4-2*, the two grapevine orthologs of *AtLYK4*, in chitosan- and chitin-induced immune responses. Functional complementation of the *atlyk4/5* mutant revealed that *VvLYK4-2*, but not *VvLYK4-1*, participates in chitosan- and chitin-mediated defense responses. Consistently, CRISPR-Cas9–mediated knock-out of *VvLYK4-2* in grapevine further highlighted its critical role in the chitosan-triggered immunity in *V. vinifera*.

## Results

### Expression profile of the grapevine orthologs of AtLYK4

**Figure 1:**
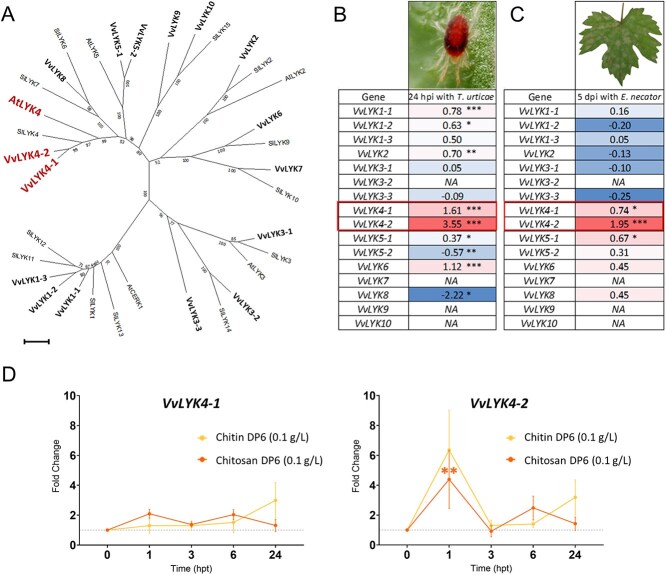
LysM-RLK phylogenetic analysis and expression profiles in *Vitis vinifera* challenged with different bioagressors or chitooligosaccharide elicitors. (A) A maximum likelihood phylogenetic tree was constructed using the Jones–Taylor–Thornton (JTT) model with 1000 bootstrap replicates in MEGA X, including LysM-RLK protein sequences from three plant species. The 16 LysM-RLKs identified in the grapevine genome (VvLYKs) are shown in bold, with VvLYK4-1, VvLYK4-2, and AtLYK4 highlighted in red. Protein ID used for this analysis are listed in Table S1. (B, C) *VvLYKs* expression profiles from *V. vinifera* leaves following inoculation with (B) *Tetranychus urticae* or (C) *Erysiphe necator.* Data represent differentially expressed genes [log_2_(fold-change)] derived from publicly available datasets of Díaz-Riquelme *et al.* [25] and Amrine *et al*. [26], respectively, and processed using the GREAT application. Harvest times after inoculation are specified in the tables. Asterisks indicate statistically differentially expressed genes (FDR-corrected *P* values adjusted to mean inoculated/non-inoculated normalized counts; ^*^P < 0.05, ^**^P < 0.01, ^***^P < 0.001). (D) Transcript accumulation of *VvLYK* genes after chitin and chitosan exposure in grapevine cells. Data represent fold change in gene expression of *VvLYK4-1 and VvLYK4-2* measured by RT-qPCR 1, 3, 6, and 24 h post-treatment (hpt) with chitin or chitosan hexamers (0.1 g/l) of *V. vinifera* cv. Marselan cell suspensions. Data represent the normalized mean fold-change ± SE from five independent experiments. Asterisks indicate a statistically significant difference to the water control (T0), set to 1 (Dunn test; ^**^P < 0.01 after ‘Bonferroni’ *P*-value adjustment). *T. urticae* cropped picture is from G. San Martin (CC BY-SA 2.0 *via* Wikimedia Commons).

In the *V. vinifera* genome, 16 genes encoding LysM-RLKs were identified, including two orthologs of *AtLYK4*: *VvLYK4-1* and *VvLYK4-2* (Fig. 1A, Table S1). As both genes are expressed in grapevine leaves (Fig. S1; [27]), we investigated if their expression was modulated by different bioaggressors: the mite *Tetranychus urticae*, the fungus *Erysiphe necator*, and the oomycete *Plasmopara viticola*, using publicly available expression datasets. Whereas *VvLYK4-1* was slightly but significantly up-regulated in leaf tissues in response to the three bioaggressors, *VvLYK4-2* was clearly upregulated in response to the first two chitin-containing organisms but not by *P. viticola* (Fig. 1B and C; Fig. S2). Additionally, to determine whether these two genes could be up-regulated by chitooligosaccharides (COS), we treated grapevine cell suspensions with purified chitin or chitosan oligomers of degree of polymerization 6 (DP6) and monitored their expression over 24 h. The real-time quantitative reverse-transcription polymerase chain reaction (RT-qPCR) experiment revealed that only the transcripts of *VvLYK4-2* were more abundant 1 h post-treatment with both COS, this accumulation being statistically significant with chitosan DP6 (Fig. 1D).

**Figure 2:**
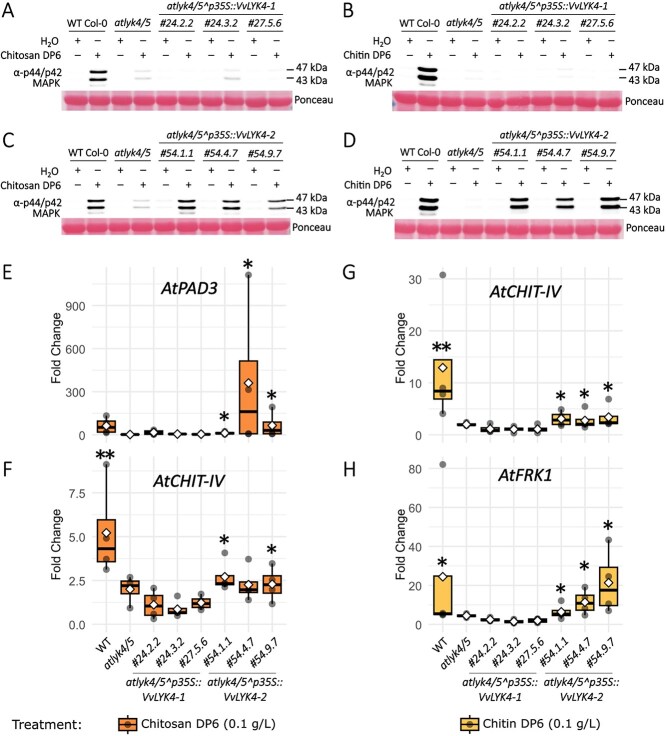
*VvLYK4-2* restores chitosan- and chitin-induced MAPK activation and defense genes expression in the Arabidopsis *atlyk4/5* double mutant. The phosphorylation of MAPKs was detected 10 min after (A, C) chitosan treatment (0.1 g/l) or (B, D) chitin treatment (0.1 g/l) by immunoblotting with an antibody raised against the human phosphorylated extracellular regulated protein kinase 1/2 (Erk1/2). Equal protein loading was confirmed by Ponceau S red staining. Similar results were obtained in three independent experiments. Fold change in gene expression of (E) *Phytoalexin deficient 3 (AtPAD3; AT3G26830),* (F, G) *Chitinase class IV (AtCHIT-IV; AT3G54420),* or (H) *Flagellin-induced receptor kinase 1 (AtFRK1; AT2G19190)* measured by RT-qPCR 1 h after chitosan (0.1 g/l) or chitin (0.1 g/l) treatment. Boxplots represent fold-change from four independent experiments compared to water control treatment set to 1. Means of these four independents experiments were represented with white diamonds. Asterisks indicate a statistically significant difference with the water control (Kruskal–Wallis with Dunn *post hoc* test; ^*^P <0 0.05, ^**^P <0 0.01, after BH *P* value adjusted). WT Col-0, wild type Columbia-0 ecotype, *atlyk4/5* double mutant for *AtLYK4* and *AtLYK5, atlyk4/5^p35S::VvLYK4-1,* double mutant *atlyk4/5* constitutively expressing *VvLYK4-1* (three independent lines obtained), *atlyk4/5^p35S::VvLYK4-2,* double mutant *atlyk4/5* constitutively expressing *VvLYK4-2* (three independent lines obtained).

### VvLYK4-2 but not VvLYK4-1 restores chitosan- and chitin-induced early immune responses in the *Arabidopsis*  *atlyk4/5* double mutant

After cloning the *VvLYK4-1* and *VvLYK4-2* coding sequences from *V. vinifera*, they were expressed into Arabidopsis *atlyk4/5* double mutant (Fig. S3) to assess whether they could complement its COS-unresponsive phenotype. The constitutive expression of *VvLYK4-1* in the double mutant *atlyk4/5* was not sufficient to restore either the chitosan- or the chitin-mediated MAPK phosphorylation in any of our three independent lines (Fig. 2A and B). Nevertheless, after the transformation of *atlyk4/5* with *VvLYK4-2,* we clearly observed in all lines the phosphorylation of the two MAPKs following both chitosan and chitin DP6 treatments (Fig. 2C and D). These results indicate that *VvLYK4-2* is sufficient to restore the MAPK phosphorylation in response to treatment with chitosan and chitin hexamers in *atlyk4/5*.

**Figure 3:**
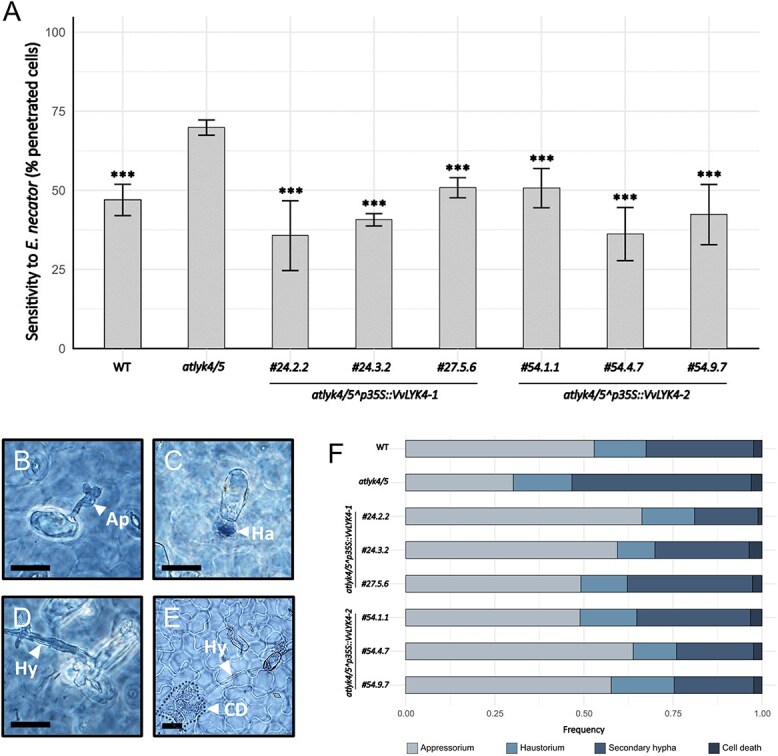
*VvLYK4-1* and *VvLYK4-2* restore penetration resistance against the non-adapted powdery mildew *E. necator* in the *atlyk4/5* double mutant. (A) Epidermal cells penetration of the non-adapted powdery mildew pathogen *E. necator* on Arabidopsis WT (Col-0), *atlyk4/5* double mutant, and three independent lines of *atlyk4/5* constitutively expressing *VvLYK4-1* or *VvLYK4-2.* One hundred germinated conidia were scored on two plants per line for each experiment. Each data point represents the mean of three independent experiments ± SE. WT Col-0 and transgenic lines were compared to the double mutant *atlyk4/5* (pairwise comparison of proportions; ^**^P <0.01 after holm *P* value adjustment). (B–E) Trypan blue staining was used to visualize fungal structures: appressorium (B), haustorium (C), secondary hypha (D), and cell death (E). Scale bar: 20 μm. Ap, appressorium; Ha, haustorium; Hy, secondary hypha; CD, cell death. (F) Detailed scoring of the barplot presented in (A). For scoring, only germinated spores were taken into account. Among these, spores that developed only an appressorium were considered unsuccessful in penetrating plant cells. In contrast, spores that formed a haustorium, secondary hyphae, or triggered cell death were regarded as having successfully penetrated the cells. Each rating reflects the most advanced stage of infection observed for the scored conidia.

To know if this MAPK phosphorylation was also coupled with defense gene expression, we measured the transcript accumulation of defense-related genes in the *A. thaliana* lines after a treatment with the two COS. Since the *phytoalexin deficient 3* (*AtPAD3*) gene encoding the last enzyme of the camalexin biosynthesis was previously found to be significantly induced in response to a chitosan treatment [18], we evaluated the accumulation of its transcripts in our different lines after exposure to chitosan DP6. While it was not up-regulated by a chitosan treatment in *atlyk4/5* or the three *atlyk4/5^p35S::VvLYK4-1* lines (#24.2.2, #24.3.2, and #27.5.6), a significant increase of *AtPAD3* transcript accumulation was detected 1 h after the treatment in the three *atlyk4/5^p35S::VvLYK4-2* lines (*atlyk4/5^p35S::VvLYK4-2* #54.1.1, #54.4.7, and #54.9.7) (Fig. 2E). A slight but significant increase in the *Chitinase-IV* transcripts was also observed after a chitosan treatment in two out of three *atlyk4/5^p35S::VvLYK4-2* lines (#54.1.1, and #54.9.7) (Fig. 2F). Transcripts of this gene were also found slightly more accumulated after a chitin treatment in the three *atlyk4/5^p35S::VvLYK4-2* lines but not in the *VvLYK4-1* expressing lines (Fig. 2G). As reported in different studies, the *Flagellin-induced receptor kinase 1* (*AtFRK1)* gene is significantly up-regulated in response to chitin treatment in the WT [9, 24], but not in the *atlyk4/5* double mutant (Fig. 2H). *AtFRK1* transcript accumulation after a chitin DP6 treatment could be restored after the transformation of *atlyk4/5* with *VvLYK4-2* in all three independent lines but not after the transformation with *VvLYK4-1* (Fig. 2H). Taken together, these results indicate that *VvLYK4-2*, unlike *VvLYK4-1*, can complement chitosan- and chitin-triggered immune responses in the *atlyk4/5* double mutant.

### VvLYK4-2 and VvLYK4-1 expression restores penetration resistance against the non-adapted powdery mildew in the *Arabidopsis*  *atlyk4/5* double mutant

We have previously demonstrated that some *VvLYK* genes can induce an increased resistance in *A. thaliana* against the non-adapted grapevine powdery mildew *E. necator* [9, 24]. We therefore investigated whether *VvLYK4-1* and *VvLYK4-2* are also important for non-host resistance to powdery mildew penetration. As revealed in our previous publication [24], the *Arabidopsis atlyk4/5* double mutant is more susceptible to *E. necator* than the WT (Fig. 3A), based on the assessment of the different fungal penetration stages (Fig. 3B–F). After the transformation of this line with the two grapevine orthologs of *AtLYK4*, we observed significantly fewer penetrated epidermal cells in all three *atlyk4/5* lines expressing *VvLYK4-2* compared to the *atlyk4/5* double mutant (Fig. 3A). This suggests that *VvLYK4-2* might be important for grapevine defense against *E. necator*. Remarkably, this restored resistance to the Arabidopsis non-adapted pathogen was also found in all three independent transgenic lines *atlyk4/5^p35S::VvLYK4-1* (Fig. 3A) whereas the *VvLYK4-1* gene could not restore the COS-induced immune responses in the *atlyk4/5* double mutant (Fig. 2).

### The grapevine *vvlyk4-2* mutants are defective in chitosan-triggered immune responses

**Figure 4:**
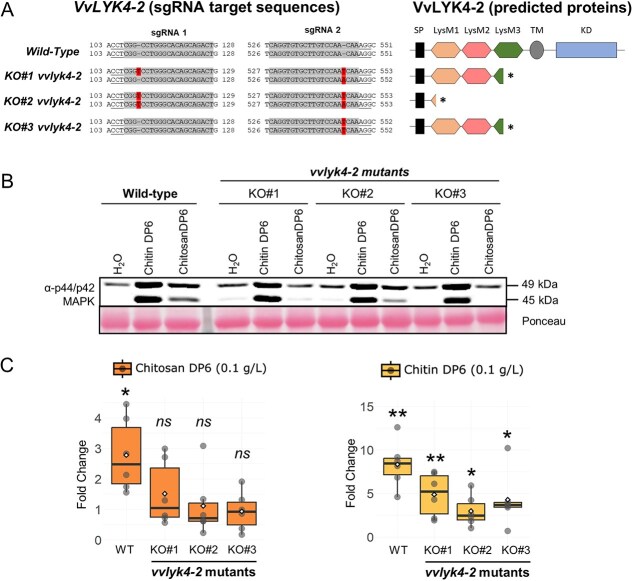
*vvlyk4-2* mutants are defective in chitosan-triggered MAPK phosphorylation. (A) Sequence analysis of three independent *vvlyk4-2* knockout lines. On the left, alignment of the two *VvLYK4-2* sgRNA target sequences used for each mutant compared to the WT. On the right, the corresponding predicted VvLYK4-2 proteins. Different domain characterizing the LysM-RLK VvLYK4-2 are represented. Black square: Signal peptide (SP), hexagon: LysM domains (LysM1-3), grey circle: transmembrane domain (TM) and blue square: kinase domain (KD). (B) Representative immunoblotting of phosphorylated MAPKs detected 10 min after H_2_0, chitin DP6 and chitosan DP6 treatment (0.1 g/l) detected with an antibody raised against the human phosphorylated extracellular regulated protein kinase 1/2 (α-pERK1/2) in the WT and three *vvlyk4-2* mutants. (C) Quantification of the MAPKs phosphorylation at 10 min after treatment, detected by ImageQuant. Boxplots represent MAPK phosphorylation level of six independent experiments each represented with grey dot. Means of these six independents were represented with white diamonds. For each experiment, leaves of three different plantlets were sampled. Asterisks indicate a statistically significant difference between treatment and the water control set to 1 for each genotype (Wilcoxon test; ^*^P <0.05, ^**^P <0.01, *ns*, non-significant).

To further investigate the role of VvLYK4-2 in chitosan- and chitin-triggered immunity in grapevine, we generated three independent knock-out (KO) lines of *V. vinifera* cv. Chardonnay using the CRISPR/Cas9 technology. The obtained mutants, named *vvlyk4-2* KO #1, #2, and #3, exhibit distinct types of mutations, each potentially resulting in different truncated proteins without any transmembrane or kinase domain (Fig. 4A, Fig. S4).

Building on the previous findings in *A. thaliana*, we analyzed the early defense responses triggered by chitosan and chitin DP6 in the grapevine mutants. The water (H_2_O) treatment induced only a slight phosphorylation of the 49 kDa MAPK in all the lines. Interestingly, the significant increase in MAPK phosphorylation induced by chitosan in WT plantlets was lost in the *vvlyk4-2* mutant lines (Fig. 4B and C). In contrast, chitin-induced MAPK phosphorylation was still detected in the three *vvlyk4-2* mutant lines (Fig. 4B and C). These results indicate that VvLYK4-2 plays a major role in chitosan-induced immunity in grapevine but may be dispensable for chitin-triggered immunity.

**Figure 5:**
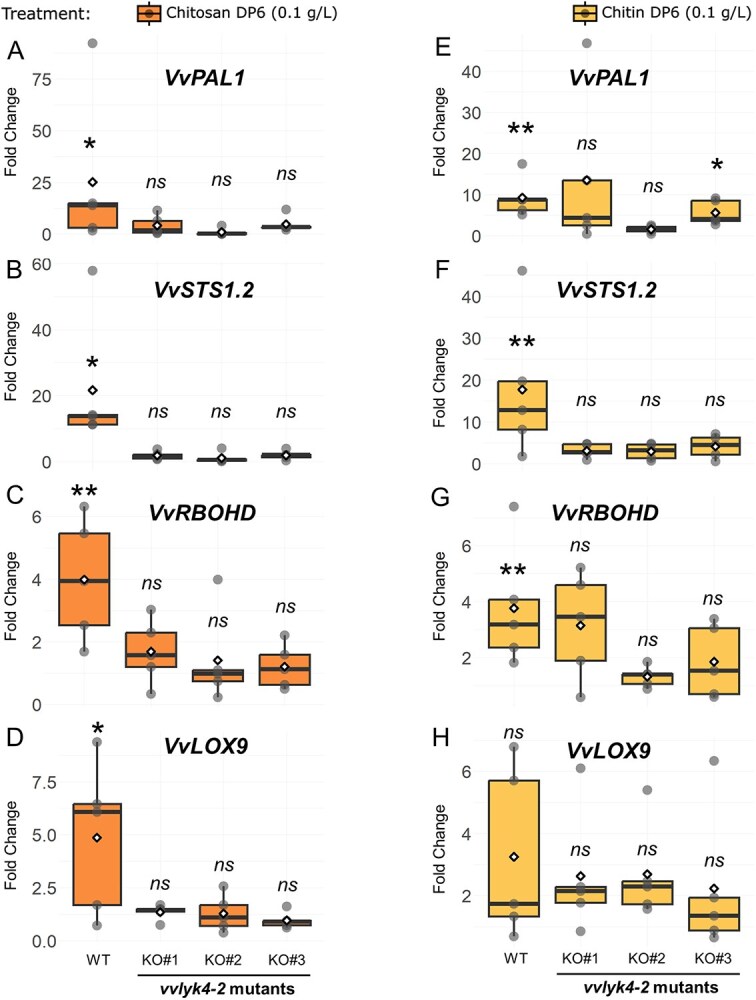
Grapevine mutants *vvlyk4-2* are defective in chitosan- and chitin-triggered defense gene expression. Gene expression of (A, E) *VvPAL1 (Vitvi00g04609),* (B, F) *VvSTS1.2 (Vitvi16g01485),* (C, G) *VvRBOHD (Vitvi01g01803),* and (D, H) *VvLOX9 (Vitvi14g00234)* measured by RT-qPCR 3 h after (A–D) chitosan (0.1 g/l) of (E–H) chitin (0.1 g/l) treatment. Boxplots represent fold change of five independent experiments. Means of these five independent experiments were represented with white diamond. For each sample, leaves of three different plantlets were sampled. Asterisks indicate a statistically significant difference between treatment and the water control set to 1 for each genotype (Wilcoxon test; ^*^P <=0.05, ^**^P <0.01, ns, non-significant).

To further confirm the role of VvLYK4-2 in chitosan-triggered immunity, we also analyzed the expression of defense genes known to be upregulated in grapevine after a chitosan DP6 treatment. Those genes encode a phenylalanine ammonia lyase *(VvPAL1)*, a stilbene synthase *(VvSTS1.2),* a respiratory burst oxidase homolog D *(VvRBOHD)*, and the oxylipin biosynthesis gene *VvLOX9* , which can be induced by a fungal infection or following an elicitor treatment [28]. In the WT, *VvPAL1*, *VvSTS1.2, VvRBOHD*, and *VvLOX9* are all induced by chitosan DP6 (Fig. 5A–D). However, no significant increase in the transcript number of these genes was observed in the three *vvlyk4-2* mutant lines after a chitosan DP6 treatment, indicating that these lines lacking a functional VvLYK4-2 receptor fail to properly activate chitosan-mediated defense gene expression (Fig. 5A–D).

Finally, we tested the induction of the same defense genes after a chitin treatment. In the WT, only *VvLOX9* was not differentially expressed in response to chitin DP6 (Fig. 5H). For *VvSTS1.2 * and *VvRBOHD*, no statistically significant increase in the transcripts of these genes was observed after a chitin treatment in the three KO lines compared to the water-mock treatment (Fig. 5F and G). Finally, the results obtained for *VvPAL1* were unclear since one of the three KO lines still responded to chitin DP6 (Fig. 5E). Considering this last result, it therefore appears difficult to stand on the importance of VvLYK4-2 in chitin-related immune responses probably due to the presence of a functional VvLYK5-1 receptor. Taken together, our results indicate that *VvLYK4-2* is a key player in chitosan-triggered immune responses but appears dispensable for chitin-mediated defense responses in *V. vinifera*.

### VvLYK4-2 has more affinity for chitosan oligomers than VvLYK4-1

**Figure 6:**
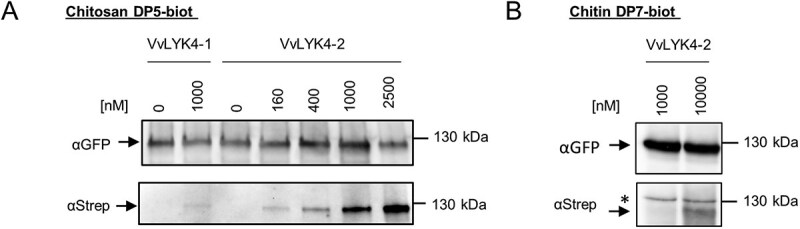
The LysM-RLK VvLYK4-2 binds chitosan with a higher affinity than VvLYK4-1. Microsomal fractions from *N. benthamiana* leaves expressing VvLYK4-1YFP and VvLYK4-2YFP were incubated with (A) a cross-linkable biotinylated chitosan DP5 or (B) with a cross-linkable biotinylated chitin oligosaccharide of DP7. The proteins were enriched using anti-GFP beads. Western blot analyses were carried out with α-GFP and streptavidin-HRP to detect the presence of the receptor proteins and the ligand bound, respectively. Western blots are representative of experiments performed with two independent batches of membrane fractions. The arrows indicate the position of the fusion proteins, whereas the asterisk indicates a nonspecific band (endogenously biotinylated protein) observed at ~130 kDa.

VvLYK4-1 and VvLYK4-2 were expressed as YFP fusion proteins in leaves of *Nicotiana benthamiana*. Membrane fractions extracted from the corresponding *N. benthamiana* leaves were used in ligand-binding assays with a crosslinkable biotinylated chitosan oligomer DP5 (chitosan DP5-biot) or chitin oligomer DP7 (chitin DP7-biot). Using the chitosan DP5-biot ligand, a signal was detected on VvLYK4-2 at a ligand concentration starting from 160 to 2500 nM (Fig. 6A). Using 1 μM (1000 nM), a stronger signal was detected on VvLYK4-2 than on VvLYK4-1 (Fig. 6A). The weaker signal detected on VvLYK4-1 may be non-specific, as it was also detected on VvLYK5 and VvLYK6 (Fig. S5). A signal was also detected for VvLYK4-2 using the chitin DP7-biot ligand (Fig. 6B), although it was only detected at a ligand concentration of 10 μM (10 000 nM), suggesting a rather low affinity for chitin oligomers. Overall, these results suggest that VvLYK4-2 has a higher affinity for chitosan oligomers than for chitin oligomers, and that VvLYK4-2 has a higher affinity for chitosan oligomers than VvLYK4-1, which is consistent with the complementation data.

## Discussion

In susceptible grapevine (*V. vinifera*), chitosan hexamers confer a strong induced resistance allowing a reduction of ~70% in the lesion diameter of *B. cinerea*, and a reduction greater than 90% in the sporulating area of *P. viticola* [9, 19]. Whereas good protection levels against grapevine pathogens can be obtained in controlled conditions, the chitosan efficacy is lower in vineyards, thus preventing the generalization of this plant protection product allowed in organic farming [19]. To understand the reason for this lack of effectiveness, it is important to better understand the mechanisms allowing the perception of chitosan by grapevine cells.

In a previous publication, we reported that the grapevine receptors VvLYK1-1 and VvLYK1-2 mediate immune responses after treatment with chitosan oligomers [9]. More recently, we discovered that the *atlyk4/5* double mutant was lacking chitosan-induced defense responses, and that neither *VvLYK5-1* nor *VvLYK5-2* was able to restore it, despite the restoration of the chitin-induced defense responses by *VvLYK5-1* [24]. Thus, we hypothesized that the perception of the deacetylated chitosan oligomers could involve one of the two grapevine orthologs of *AtLYK4*. According to gene expression data, *VvLYK4-1* and *VvLYK4-2* were up-regulated by different bioaggressors such as mites or fungi, known to be sources of COS. After the transformation of *VvLYK4-1* or *VvLYK4-2* in the *atlyk4/5* double mutant, we showed that *VvLYK4-2*, but not *VvLYK4-1*, was sufficient to restore early defense responses, including MAPK phosphorylation and defense gene expression in response to both deacetylated chitosan and acetylated chitin oligomers. To validate these results and evaluate the importance of the *VvLYK4-2* gene in COS-mediated immune responses, we then generated three independent *vvlyk4-2* knock-out lines in grapevine. Whereas both MAPK activation and defense gene expression following a chitosan treatment were clearly and strongly altered by the *vvlyk4-2* knock-out, immune responses appeared less impaired in these mutant lines after a treatment with chitin hexamers. Indeed, while the MAPK activation was still observed in the mutant lines treated with chitin DP6, only part of the defense genes monitored showed a strong and significant reduction in their expression in the knock-out lines. This is consistent with the ligand-binding assays suggesting that VvLYK4-2 has higher affinity for chitosan oligomers than chitin oligomers and with the presence in the mutant lines of a *VvLYK5-1*/*VvLYK1-1* couple known to be involved in the chitin-induced defense responses, at least for some immune signaling pathways.

Taken together, our results reveal that VvLYK4-2 plays a crucial role in the defense responses induced by chitosan and immunity. *VvLYK4-1* is also up-regulated in response to different bioaggressors and interestingly, its constitutive expression in *atlyk4/5* double mutant also restores the penetration resistance to the non-adapted powdery mildew *E. necator*. VvLYK4-1 is not able to restore the COS-induced early defenses, suggesting that it is not involved in COS signaling or require the presence of VvLYK5 as proposed for AtLYK4 and AtLYK5. Notably, protein sequence comparison between VvLYK4-1 and VvLYK4-2 revealed amino acid differences within the LysM2 domain, a key domain implicated in COS perception (Fig. S6). Such variations could affect ligand binding or receptor complex formation [29] that might explain the functional divergence between VvLYK4-1 and VvLYK4-2. As the increased resistance to *E. necator* in *atlyk4/5* expressing *VvLYK4-1* is not linked to COS-induced early defenses, we hypothesize that this receptor might be also involved in the perception of other danger signals, which could be released after the degradation of cell walls during fungal penetration in its attempt to colonize plant tissues.

Phylogenetic analysis suggests that a tandem duplication occurred in the ancestor of VvLYK4-1 and VvLYK4-2 before the emergence of eudicotyledons. Indeed, the two genes are well conserved in most eudicotyledons, although one of the two genes was lost in legumes and *Brassicaceae* [30]. In *A. thaliana*, *AtLYK4* is the closest ortholog of *VvLYK4-2* and the chitosan perception is altered in the *atlyk4/5* double mutant, suggesting that AtLYK4 might participate to the chitosan-induced defense responses. As the extracellular domain of this receptor has been expressed and purified from insect cells [16], it would be worth testing the binding of chitosan by AtLYK4, which has not been done before, as it was restricted to chitin and laminarihexaose.

Recently, a LysM-RLK from *Glycine max* GmNFR5a, belonging to another phylogenetic group, was proposed to be involved in chitosan-triggered immunity [31]. The extracellular domain of GmNFR5a has been expressed and purified from *Pichia pastoris* and showed rather low affinities for chitin and chitosan oligomers (mM range), which is consistent with the fact that this group of LysM-RLK is well known to be involved in Nod factor perception, a chitin derivative containing the acyl chain [30].

A more systematic determination of the LysM-RLK affinities for chitosan is thus required to depict how plants perceive chitosan. Unfortunately, the crosslinkable chitosan DP5-biot we have used provides qualitative information on chitosan binding, but shows some non-specific binding, which makes difficult to determine receptor affinities for chitosan. The development of other chitosan derivatives or binding assays is therefore required to enable an accurate comparison of affinity between candidate receptors.

Identifying chitosan receptors will also help to understand how signaling mechanisms differ following chitosan and chitin perception. In *A. thaliana*, fully deacetylated chitosan oligomers did not induce any production of ROS whereas it can be detected with chitin of the same DP and concentration [32]. The accumulation of *PAD3* transcripts in response to chitosan oligomers revealed by Povero *et al*. [18] appears to be chitosan-specific as no significant change in its gene expression could be detected in response to a chitin treatment (Fig. S7). Similarly, in grapevine, *LOX9* transcripts significantly accumulated only after a treatment with chitosan oligomers. Understanding how the perception of these closely related molecules can lead to different defense responses is thus a challenging question for further studies.

Finally, exploring the genetic variability between *Vitis* sp. or *V. vinifera* cultivars may help to better understand chitosan perception. If the efficacy of chitosan-based biocontrol products differs according to the *Vitis* genotypes, it would be interesting to see if single mutations or larger polymorphisms present in the *VvLYK* sequences could impact chitosan perception by the plant. The identification of natural mutations existing within chitosan-unresponsive *Vitis* sp. or *V. vinifera* cultivars could reveal key residues involved in the binding of chitosan or cis-elements involved in the transcriptional regulation of the candidate receptors. Similarly, if the expression of *VvLYK1-1/1-2/4-2* genes is low in susceptible organs such as young leaves or inflorescences, finding a way to enhance their expression before a chitosan treatment could enhance the chitosan efficacy in protecting the plant against several diseases.

## Materials and methods

### Expression analysis of VvLYK genes in response to biotic stresses

Expression data for *VvLYK* genes were retrieved from public RNAseq datasets available on the NCBI platform (https://www.ncbi.nlm.nih.gov/) using the *GRape Expression ATlas (GREAT)* tool (https://great.colmar.inrae.fr/). *VvLYK* expression profiles were obtained from different studies: *Vitis vinifera* cv. Tempranillo leaves 24 h post-inoculation with *T. urticae* are from the BioProject Accession PRJNA293344 [25], *V. vinifera* cv. Carignan leaves 5 days post-inoculation with *E. necator* are from the BioProject Accession PRJNA279229 [26], and *V. vinifera* cv. Pinot Noir leaves 24 h post-inoculation with *P. viticola* are from the BioProject Accession PRJNA168987 [33].

### Plant materials and elicitors

*Arabidopsis thaliana* ecotype Columbia (Col-0), the double mutant *atlyk4/5* (WiscDsLox297300_01C x SALK_131911C; [11]), and transgenic lines were cultivated at 20°C/18°C under a 10-/14-h day/night cycle. Transgenic lines were generated by floral-dip [34] using the pFAST_R02 overexpression vector [35] harboring coding sequences of *VvLYK4-1* or *VvLYK4-2* amplified from complementary DNA (cDNA) derived from *V. vinifera* cv. Marselan leaves. Three independent homozygous lines were obtained for each construct (i.e. *atlyk4/5^p35S::VvLYK4-1* and *atlyk4/5^p35S::VvLYK4-2*; Fig. S3).

Suspension cultures of *V. vinifera* cv. Marselan cells were grown and subcultured in Nitsch-Nitsch liquid medium [36] as described by Roudaire *et al*. [24]. Cells used in experiments were collected after a 1:2 dilution and 24 h of growth of the previous culture.

Embryogenic calli of grapevine (*V. vinifera* cv. Chardonnay) were transformed by co-cultivation with *Agrobacterium tumefaciens* strain EHA105 carrying the *VvLYK4-2z_pDGB3α1* construct. This latter was assembled using the GoldenBraid cloning method [37] and comprises a *Zea mays* codon-optimized Cas9 with Arabidopsis introns and two nuclear localization signals (zCas9i; [38]) under the control of the 35S promoter. It also includes a multiplexing cassette [39] of three sgRNAs targeting *VvLYK4-2* and two plant selection markers, namely *NPTII* and *DsRed2*. SgRNAs were designed with CRISPOR (https://crispor.gi.ucsc.edu/; [40]) and recommendations from other studies [41, 42] to be specific to *VvLYK4-2*. We checked by sequencing the genomic region of the off-target exon with the highest risk score (CFD > 0.1) and detected no mutations in the selected lines (Fig. S8). Following somatic embryogenesis and plantlet regeneration, CRISPR-Cas9-edited mutants for *VvLYK4-2* were cultivated in McCown medium (M0220, Duchefa Biochemie) supplemented with 30 g/l sucrose and 0.5 mg/l indole butyric acid under a 16/8-h day/night cycle at 26°C. The experimental procedure used to select and generate the three independent *VvLYK4-2* mutant lines was carried out similarly to Villette *et al*. [43].

Purified chitin (*GLU436*) and chitosan (*GLU426*) hexamers (DP6) were kindly provided by Elicityl (Crolles, France), dissolved in ultrapure water, and used at 0.1 g/l for all treatments.

### MAPK activation assays

Leaves from 4-week-old Arabidopsis or 6-week-old grapevine plants were detached, pre-infiltrated with ultrapure water, and equilibrated (abaxial face down) on ultrapure water for 4 h in a 6-well plate. They were subsequently treated by substitution of water with elicitors or water (mock treatment) and collected 10 min later. *Arabidopsis thaliana* proteins were extracted as described by Roudaire *et al*. [24]. *Vitis vinifera* proteins were extracted using TRI reagent (Sigma-T9424), phase-isolated with 1-bromo-3-chloropropane, and precipitated with 100% acetone. Phosphorylated MAPKs were detected by western blotting using the anti-p42/44-phospho-ERK antibody (1:5000; Cell Signaling) and a horseradish peroxidase-conjugated goat anti-rabbit IgG secondary antibody (1:20 000; Agrisera). Revealing was carried out on an Amersham™ ImageQuant™ 800 system (Cytiva) using ECL™ Prime detection reagent. Ponceau staining was used to check transfer and loading quality. Experiments were independently repeated three times.

### Real-time quantitative reverse-transcription polymerase chain reaction

*A. thaliana* leaves were harvested 1 h post-treatment (hpt) under conditions identical to MAPK assays and grapevine cell suspensions were collected at 1, 3, 6, and 24 hpt. Total RNAs were extracted using the SV Total RNA Isolation System (Promega) with on-column DNAse. Reverse transcription step, qPCR reactions and data analysis based on the Common Base Method [44] and LinRegPCR corrections [45] were performed as previously described [24]. *AtPTB1* (*AT3G01150*)/*AtRHIP1* (*AT4G26410*) or *VvVATP16* (*Vitvi03g04022*)/*VvRPL18B* (*Vitvi05g00033*)/*VvVPS54* (*Vitvi10g01135*) were used as housekeeping genes for Arabidopsis or grapevine, respectively.

Grapevine *in vitro* plantlets treatments also mirrored those for MAPK assays but leaves were harvested 3 h post-treatment. RNA isolation was done with the Macherey-Nagel™ RNA Plant and Fungi NucleoSpin kit. The qPCR analysis also followed the same procedure as above using *VvVATP16* (*Vitvi03g04022*) and *VvEF1alpha* (*Vitvi06g04107*) as housekeeping genes.

Experiments were independently repeated at least four times. Primers used are listed in Table S2. Melting curves of the different qPCR realized in this study are presented in Fig. S9.

### *Erysiphe necator* infection assay

*Erysiphe necator* cultures were maintained on leaves of *V. vinifera* cv. Marselan surface-sterilized using a solution of 2% (v/v) sodium hypochlorite, and refreshed biweekly on sterile agar plates. Four-week-old Arabidopsis plants were inoculated by brushing *E. necator* spores onto two leaves per plant. Infected leaves (two plants per line) were sampled 48 h post-inoculation (hpi), stained with trypan blue [46], and examined under a Leica (Wetzlar, Germany) DME microscope at magnification ×400. Penetration success was assessed based on haustorium formation, secondary hyphae development or cell death observation [24], scoring at least 100 germinated spores per treatment. Experiments were independently repeated three times.

### Expression in heterologous system and ligand-binding assay

p35S:VvLYK4-1-YFP (this study), p35S:VvLYK4-2-YFP (this study), p35S:VvLYK5-YFP [24], p35S:VvLYK6 [47] were expressed in *N. benthamiana* leaves, and the membrane fractions were isolated as previously described in Girardin *et al.* [48]. Chitosan DP5-biot synthesis is described in Fig. S10 [49]. Chitin DP7-biot synthesis was detailed in Ding *et al*. [50]. Binding assays with Chitosan DP5-biot were conducted using 200 μg of microsomal proteins in a buffer (25 mM NaCacodylate pH 6.0, 1 mM MgCl_2_, 1 mM CaCl_2_, 250 mM saccharose, and protease inhibitors) for 1 h on ice. After incubation, samples were centrifuged at 31000 *g* for 30 min at 4°C, and the pellets were resuspended in IP buffer (25 mM Tris–HCl pH 7.5, 150 mM NaCl, 10% glycerol, supplemented with protease inhibitors and the phosphatase inhibitor). The proteins were then solubilized in the IP buffer containing 0.2% DDM for 1 h at 4°C and immunopurified using GFP-trap magnetic agarose beads (ChromoTek). Following washing in IP buffer, proteins were eluted with Laemmli buffer. Binding assays with Chitin DP7-biot were performed on using 50 μg of microsomal proteins as described above expected that after the centrifugation pellets were washed with the binding buffer and resuspended directly in Laemmli buffer. Western blots were performed using rabbit anti-GFP antibodies (1:7000; AMSBIO), followed by and HRP-conjugated goat anti-rabbit secondary antibody (1:20000, Millipore) and Streptavidin-HRP (1:10000, Sigma) to detect the presence of the receptor proteins and the ligand bound, respectively.

## Supplementary Material

Web_Material_uhag097

## Data Availability

*Vitis vinifera* cv. Marselan *VvLYK4-1* (PV383271) and *VvLYK4-2* (PV383272) sequences have been deposited to GenBank and are accessible from NCBI. The data underlying this article are available in the article and in its online supplementary material.
