# Supplementary material for: The grapevine LysM receptor kinase VvLYK4-2 is a key player in chitosan-triggered immune responses
Source: Hortic Res. 2026 Mar 13;13(7):uhag097. doi: 10.1093/hr/uhag097 (PMC13278843; doi:10.1093/hr/uhag097)
Supplement: Web_Material_uhag097 [file web_material_uhag097.zip › Roudaire et al 2025 sup Table_R2.pdf]

Table S1. Thirty four listed LysM-RLKs identified in 3 plant species were used for phylogenetic analysis in supplemental figure 1. Names of LysM-RLKs were taken from Buendia et al., 2018 and corresponding ID are detailed.

Plant species	LysM-RLK	ID
Solanum lycopersicum	SlLYK13	Solyc01g098410
Solanum lycopersicum	SlLYK11	Solyc02g081040
Solanum lycopersicum	SlLYK12	Solyc02g081050
Solanum lycopersicum	SlLYK3	Solyc03g121050
Solanum lycopersicum	SlLYK14	Solyc06g069610
Solanum lycopersicum	SlLYK1	Solyc07g049180
Solanum lycopersicum	SlLYK15	Solyc11g069630
Solanum lycopersicum	SlLYK10	Solyc02g065520
Solanum lycopersicum	SlLYK2	Solyc02g094010
Solanum lycopersicum	SlLYK9	Solyc09g083210
Solanum lycopersicum	SlLYK7	Solyc02g089920
Solanum lycopersicum	SlLYK4	Solyc02g089900
Solanum lycopersicum	SlLYK6	Solyc12g089020
Vitis vinifera	VvLYK1-1	Vitvi05_01chr12g07020
Vitis vinifera	VvLYK1-2	Vitvi05_01chr10g00720
Vitis vinifera	VvLYK1-3	Vitvi05_01chr10g00730
Vitis vinifera	VvLYK2	Vitvi05_01chr14g24160
Vitis vinifera	VvLYK3-1	Vitvi05_01chr09g03910
Vitis vinifera	VvLYK3-2	Vitvi05_01chr04g06520
Vitis vinifera	VvLYK3-3	Vitvi05_01chr01g10230
Vitis vinifera	VvLYK4-1	Vitvi05_01chr04g17160
Vitis vinifera	VvLYK4-2	Vitvi05_01chr04g17150
Vitis vinifera	VvLYK5-1	Vitvi05_01chr18g00270
Vitis vinifera	VvLYK5-2	Vitvi05_01chr18g00280
Vitis vinifera	VvLYK6	Vitvi05_01chr05g09230
Vitis vinifera	VvLYK7	Vitvi05_01chr04g17140
Vitis vinifera	VvLYK8	Vitvi05_01chr18g13530
Vitis vinifera	VvLYK9	Vitvi05_01chr17g13770
Vitis vinifera	VvLYK10	Vitvi05_01chr06g04020
Arabidopsis thaliana	AtCERK1	AT3G21630.1
Arabidopsis thaliana	AtLYK2	AT3G01840.1
Arabidopsis thaliana	AtLYK3	AT1G51940.1
Arabidopsis thaliana	AtLYK4	AT2G23770.1
Arabidopsis thaliana	AtLYK5	AT2G33580.1

Table S2. Primers used in this study.

Gene	Primer name	Sequence (5' → 3')	Usage
VvLYK4-1	VvLYK4-1_full_F	ATGGCTTGGCTATCCTTCATCTC	Cloning
VvLYK4-1	VvLYK4-1_full_R	CTAACTGGAATTTTGGTATCCTGAGAC	Cloning
VvLYK4-1	VvLYK4-1_qF	GAGAGCTTTGAGGCATGTGAGA	RT-qPCR & genotyping
VvLYK4-1	VvLYK4-1_qR	GTGCCACGGTAAACAGACCC	RT-qPCR & genotyping
VvLYK4-2	VvLYK4-2_full_F	ATGAGATCTTCCAATTCAACCAAAATGGC	Cloning
VvLYK4-2	VvLYK4-2_full_R	TTATCCTGCACCATTATTAGACGATTCCC	Cloning
VvLYK4-2	VvLYK4-2_qF	TGTGCTTCATGAGGATGACGG	RT-qPCR & genotyping
VvLYK4-2	VvLYK4-2_qR	CAGGCTTGTGAAGAGGCCAA	RT-qPCR & genotyping
zCas9oi	zCas9i_geno_FP	TTGGCACATACCATGACCTG	Genotyping
zCas9oi	zCas9i_geno_RP	ATCTGACTCCCAAGCTCCTT	Genotyping
T-DNA	LB_o8474	ATAATAACGCTGCGGACATCTACATTTT	Genotyping
T-DNA	WiscDs_LB	TCCTCGAGTTTCTCCATAATAATGT	Genotyping
AtLYK4	AtLYK4_LP	CATTTTCATCCATCGATGGAC	Genotyping
AtLYK4	AtLYK4_RP	TTCCCTTTCACAACAATCCTG	Genotyping
AtLYK5	AtLYK5_LP	CTTCTTGCCGCTCATACCTC	Genotyping
AtLYK5	AtLYK5_RP	AGCCAATCACTGATCGATCC	Genotyping
AtChit-IV	AtCHIT-IV_qF	ATTGCGGTTGTTCGTCAGAG	RT-qPCR
AtChit-IV	AtCHIT-IV_qR	GGGGAGGAGCAAAACAAGGT	RT-qPCR
AtFRK1	AtFRK1-F	TGAAGGAAGCGGTCAGATTT	RT-qPCR
AtFRK1	AtFRK1-R	CTGACTCATCGTTGGCCTCT	RT-qPCR
AtRHIP1	AtOLI_q1F	GAGCTGAAGTGGCTTCCATGA	RT-qPCR
AtRHIP1	AtOLI_q1R	CGTCCGACATACCCATGATCC	RT-qPCR
AtPTB1	atPTB1	GATCTGAATGTTAAGGCTTTTAGCG	RT-qPCR
AtPTB1	AtPTB2	GGCTTAGATCAGGAAGTGTATAGTCTCTG	RT-qPCR
AtPAD3	AtPAD3_F	GGGTACCATACTTGTTGAGATGG	RT-qPCR
AtPAD3	AtPAD3_R	TTGATGATCTCTTTGGCTTCC	RT-qPCR
VvEF1α	VvEF1alpha-Fw1	TCTGCCTTCTTCCTTGGGTA	RT-qPCR
VvEF1α	VvEF1alpha-Rv1	GCACCTCGATCAAAAGAGGA	RT-qPCR
VvVATP16	Vv_VATP16_F	CTTCTCCTGTATGGGAGCTG	RT-qPCR
VvVATP16	Vv_VATP16_R	CCATAACAACTGGTACAATCGAC	RT-qPCR
VvRPL18B	VvRPL18B_qF	CCCCTATGCTTTTTGTGGACTTG	RT-qPCR
VvRPL18B	VvRPL18B_qR	TGCTCGTTTGGGACAATAAACC	RT-qPCR
VvVPS54	VvVPS54_qF	GCTGTTTTTGCGGCTTGTGA	RT-qPCR
VvVPS54	VvVPS54_qR	ACCTTCCACCAATCTTCTCCGT	RT-qPCR
VvSTS1.2	VvSTS1.2_qF	AGGAAGCAGCATTGAAGGCTC	RT-qPCR
VvSTS1.2	VvSTS1.2_qR	TGCACCAGGCATTTCTACACC	RT-qPCR
VvPAL1	VvPAL_qF	AGTCTCCATGGACAACACCCG	RT-qPCR
VvPAL1	VvPAL_qR	TGCTCAGCACTTTCGACATGG	RT-qPCR
VvRBOHD	VvRBOHD_qF	CACCACCATGCTTCAGTCCCTCCAT	RT-qPCR
VvRBOHD	VvRBOHD_qR	AGCGATCTTCTTGAAGACTTGTCGCC	RT-qPCR
VvLOX9	VvLOX9_qF	CTGCGTGGCTTCTGCTCTC	RT-qPCR
VvLOX9	VvLOX9_qR	CCATCAATCTGCGGCTTATC	RT-qPCR
