# Supplementary material for: The grapevine LysM receptor kinase VvLYK4-2 is a key player in chitosan-triggered immune responses
Source: Hortic Res. 2026 Mar 13;13(7):uhag097. doi: 10.1093/hr/uhag097 (PMC13278843; doi:10.1093/hr/uhag097)
Supplement: Web_Material_uhag097 [file web_material_uhag097.zip › Roudaire et al 2026 sup Figures_R2.pdf]

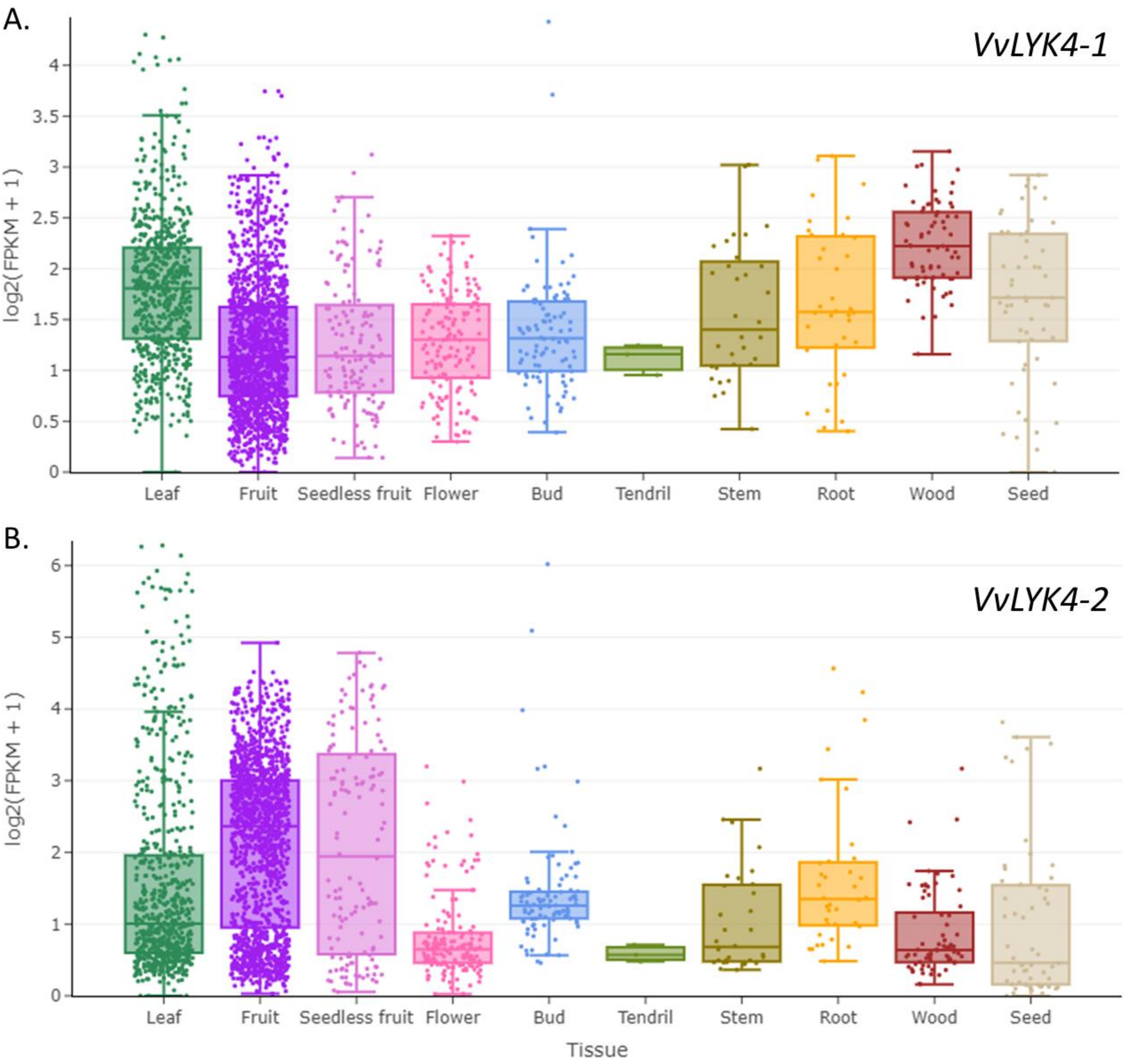

Figure S1. Basal expressions of *VvLYK4-1* and *VvLYK4-2*. (**A-B**) Data are log-transformed fragments per kilobase of exon per million mapped fragments (log_2(FPKM+1)) obtained from *Gene CARD* (http://147.156.207.74:4242/Catalogue/; Navarro-Payá et al., 2022) for (**A**) *VvLYK4-1* and (**B**) *VvLYK4-2* genes.

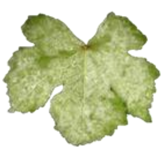

Gene	24 hpi with *P. viticola*
VvLYK1-1	0.17
VvLYK1-2	3.01 ***
VvLYK1-3	0.51 ***
VvLYK2	-0.97 ***
VvLYK3-1	1.31 ***
VvLYK3-2	*NA*
VvLYK3-3	0.30
VvLYK4-1	0.62 **
VvLYK4-2	-0.15
VvLYK5-1	-1.32 ***
VvLYK5-2	-0.74 ***
VvLYK6	0.87 ***
VvLYK7	*NA*
VvLYK8	-2.39 ***
VvLYK9	*NA*
VvLYK10	*NA*

Figure S2. LysM-RLK expression profiles in *Vitis vinifera* challenged with *Plasmopara viticola*. *VvLYKs* expression profiles from *V. vinifera* leaves following inoculation with *P. viticola*. Data represent differentially expressed genes [$\log_2$(Fold-change)] derived from publicly available datasets of Perazzolli et al. (2012) and processed using the *GREAT* application**.** Harvest time is 24h post-inoculation (hpi) with *P. viticola*. Asterisks indicate statistically differentially expressed genes (FDR-corrected p-values adjusted to mean inoculated/non-inoculated normalized counts; *, $P < 0.05$, **, $P < 0.01$, ***, $P < 0.001$).

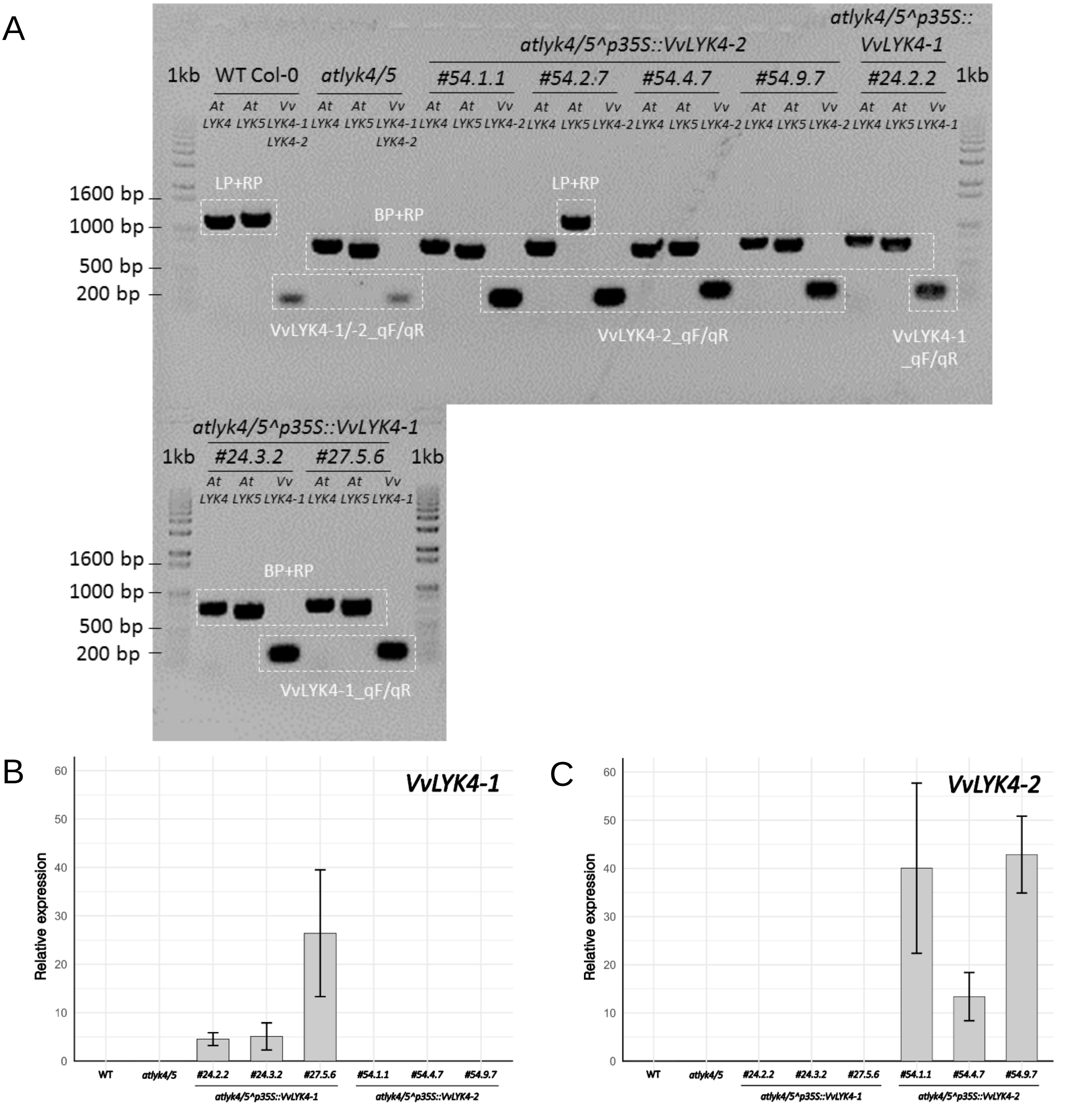

Figure S3. Characterization of the transgenic lines obtained. (**A**) Genotyping of the lines used in the study. A first polymerase chain reaction (PCR) was performed on genomic DNA (gDNA) to check the presence or the absence of the T-DNA in the wild-type (WT) or transgenic lines. The same gDNA was used in a second PCR to check the presence of the grapevine transgenes. Primers used are listed in Table S2 and have been designed using the T-DNA Primer Design tool of the SIGnAL website (http://signal.salk.edu/tdnaprimers.2.html). Expected sizes with the LP + RP primers were close to 1100 bp for the WT alleles of *AtLYK4* and *AtLYK5*, respectively. Line number *#54.2.7*, only mutated for *atlyk4*, was thus discarded for further analysis. The presence of transgenes of *VvLYK4-1* and *VvLYK4-2* in the *atlyk4/5* double mutant was checked using the “qPCR” primers used to amplify a region of approximately 200 bp in each transcript. Negative controls were performed on untransformed WT Col-0 and *atlyk4/5* double mutant. (**B-C**) Relative expression of the grapevine transgenes detected during RT-qPCR experiments. The mean of efficiency-weighted $C_q^{(w)}$ values from technical duplicates of the water control-treated samples were normalized by the $C_q^{(w)}$ average of two housekeeping genes (*AtRHIP1* and *AtPTB1*). Data represent the mean relative expression ± SE from three independent experiments.

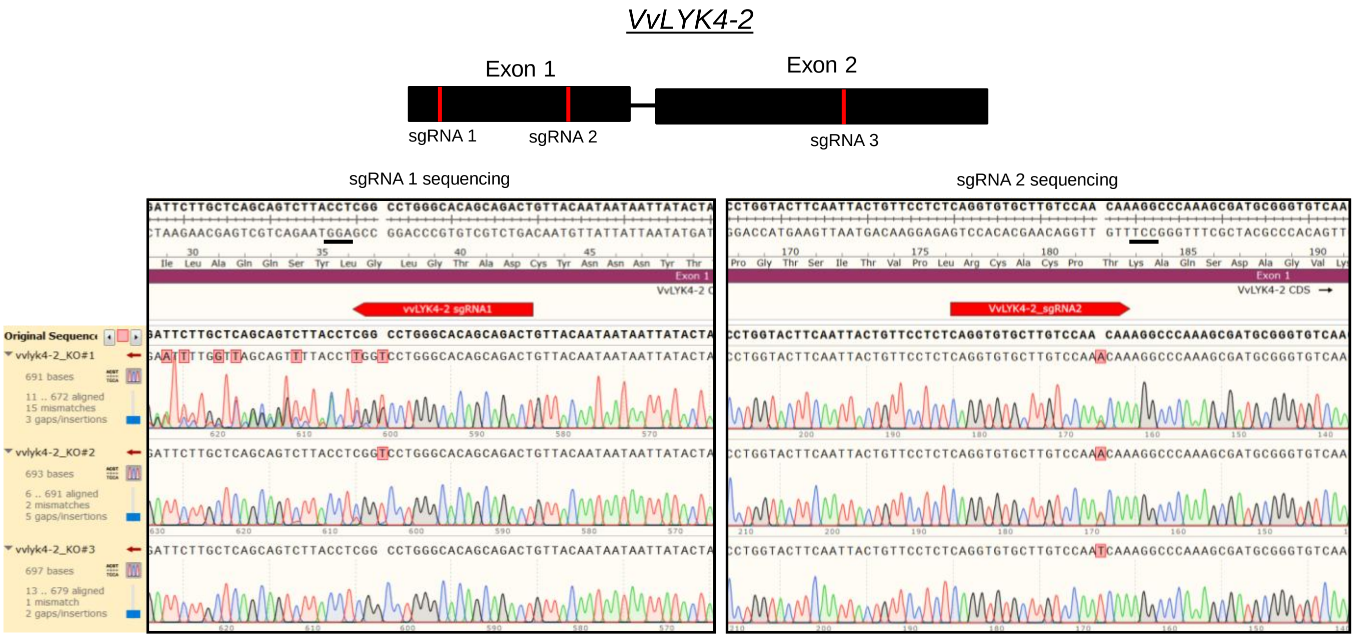

Figure S4. Characterization of CRISPR-edited lines targeting *VvLYK4-2*. Localization of the sgRNAs targeting *VvLYK4-2*. Additionally, sequencing of each line to highlight the nature of the mutations at the targeted sites for exon 1. Exon 2 was not sequenced due to the presence of an early stop codon in the selected lines, making the task unnecessary.

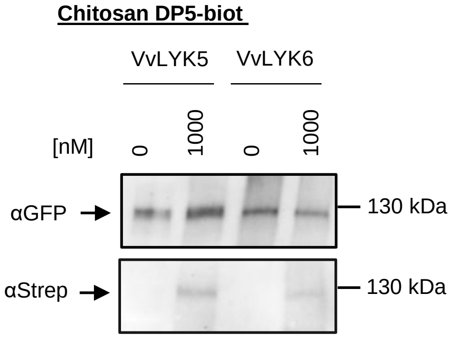

Figure S5. The LysM-RLK VvLYK5 and VvLYK6 weakly bind chitosan. Microsomal fractions from *N. benthamiana* leaves expressing VvLYK5–YFP and VvLYK6–YFP were incubated with a cross-linkable biotinylated chitosan DP5 and the proteins were enriched using anti-GFP beads. Western blot analyses were carried out with α-GFP and streptavidin–HRP to detect the presence of the receptor proteins and the ligand bound, respectively. The arrows indicate the position of the fusion proteins.

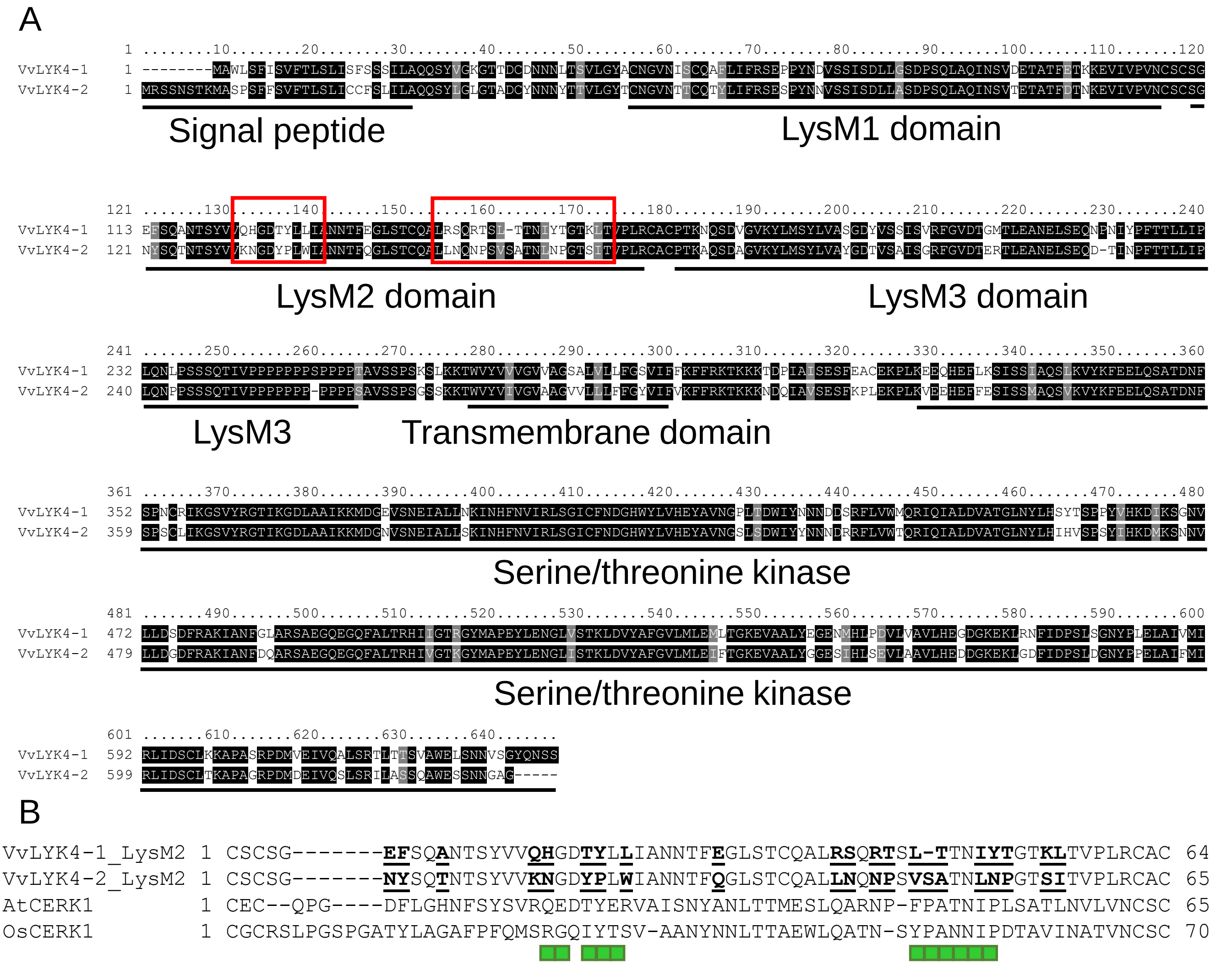

Figure S6. Identification of existing mutations in the LysM2 domain of VvLYK4-1 and VvLYK4-2. (A) Clustal alignment of VvL4-1 and VvLYK4-2 performed with MEGAX (Kumar *et al.* 2018). Amino acid conservation is highlighted using Boxshade coloring, with black indicating conserved residues and white indicating divergent residues. The known domains of the LysM receptor-like kinase are underlined in black, including the signal peptide, LysM1, LysM2, LysM3, transmembrane domain, and serine/threonine kinase domain. Regions containing multiple mutations in LysM2 domain are highlighted with red boxes. **(B)** Clustal alignment of the LysM2 domain from VvLYK4-1 and VvLYK4-2, together with the LysM2 domains of OsCERK1 and AtCERK1, was performed using MEGA X. Mutations between VvLYK4-1 and VvLYK4-2 are indicated in bold and underlined. Green squares mark the residues of AtCERK1 and OsCERK1 involved in chitin binding.

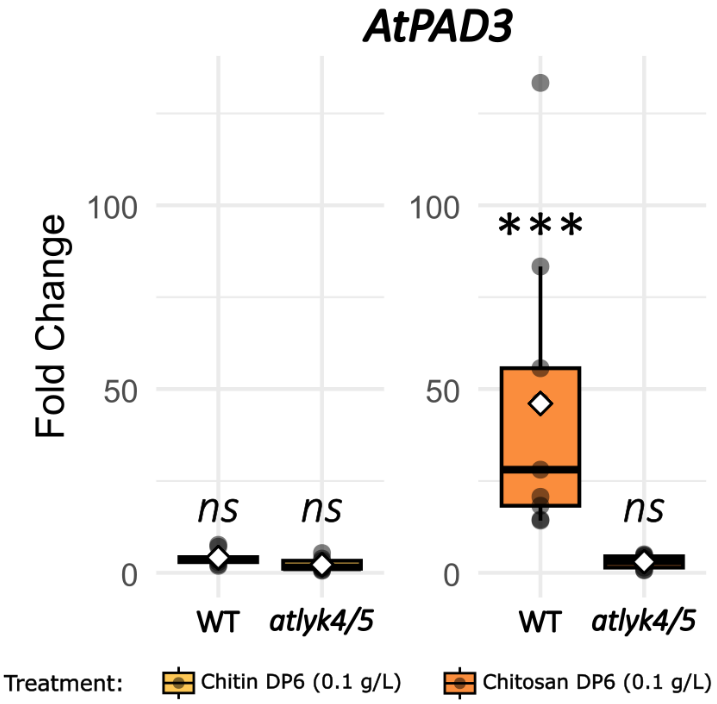

Figure S7. *Arabidopsis thaliana lyk4/5* double mutant is altered in chitosan-induced *PAD3* gene expression. Fold change in gene expression of *Phytoalexin deficient 3 (AtPAD3; AT3G26830)* was measured by qRT-PCR 1h after chitin DP6 (0.1 g/L), chitosan DP6 (0.1 g/L), or water treatment. Boxplots represent fold-change from nine independent experiments compared to water control treatment set to 1. Means of these nine independent experiments were represented with white diamond. Asterisks indicate a statistically significant difference with the water treatment (Wilcoxon test **, P < 0.01, ns, non-significant). WT, wild-type, Columbia-0 ecotype.

A

Off-target id	Guide and off-target sequence with mismatches position	CFD off-target score	Locus description
sgRNA1	GAGGCTTTGACCGGCGACAAGGG		
**Off-target1**	AGTCTGCTGTTCCCAAGCTTTGG<br>..........*....*..**	**0.124260355**	Chromosome 7 - LOC100254266-Hydrolase - Exon
Off-target2	AGCCTGCTGGGCTCGGGCCGCGG<br>..*......*..*.*.....	0.0975	Chromosome 7 intergenic region
sgRNA2	CAGGTGTGCTTGTCCAACAAAGG		
Off-target3	CATATCTGTTTGTCCAACAAAGG<br>**.....*...*........	0.268465909	Chromosome 12 intergenic region
Off-target4	TCGGTGTTCTTCTCCAACAAAGG<br>..**...*..*.........	0.259978992	Chromosome 11 intergenic region
Off-target5	AATGTGTGCTTGTTCAACATGGG<br>*.*..........*.....*	0.22	Intron of LOC100257259
Off-target6	CAGGTTTCCCTATCCAACAATGG<br>.....*.*.*.*........	0.204216524	Intron of LOC100244920
Off-target7	GAGGTGTCATTGTACAACAATGG<br>*......**....*......	0.168561873	Chromosome 7 intergenic region
Off-target8	CATGTGGGCTTGATCAACAAGGG<br>..*...*.....**......	0.149321267	Chromosome 6 intergenic region
Off-target9	CAGGTGTGCATCTCAGACAATGG<br>.........*.*..**....	0.019392372	Chromosome 14 - LOC100259498 - Exon
Off-target10	CAGGTGTGGTTATCTCACAAAGG<br>........*..*..**....	0	Chromosome 17 intergenic region
Off-target11	AAGGTGTGCCTTTCCAACAAGGA<br>*........*.*........	0.014245014	Chromosome 2 intergenic region
sgRNA3	CAGGTGTGCTTGTCCAACAAAGG		
Off-target3	GCCTTGACGAGGCACATCGTTGG<br>*.....*.......*...*.	0.043582375	Chromosome 2 - LOC100247907 - Exon
Off-target4	GCCTTGACGAGGCACATCGTTGG<br>.....*..*.....*...*.	0.025641026	Chromosome 4 - LOC100242712 - Exon

B

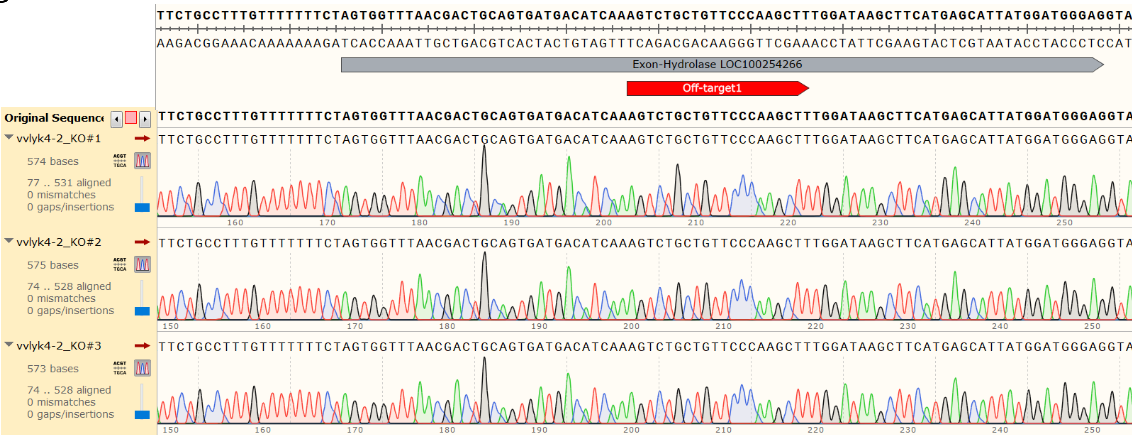

Figure S8. Off-target analysis in *vvlyk4-2* mutant lines. (**A**) Listing of predicted off-targets for both sgRNAs. Two off-targets are predicted for sgRNA1, and nine off-targets are predicted for sgRNA2. The sequence of different off-targets, the off-target risk score, and locus description are detailed in the second, third, and fourth columns, respectively. Asterisks represents mismatches between sgRNA and off-target sequences. (**B**) Off-target1 (targeting the exon of a hydrolase with a risk score > 0.1) was sequenced for the three mutant lines.

Melting curves for genes expression of Figure 1:

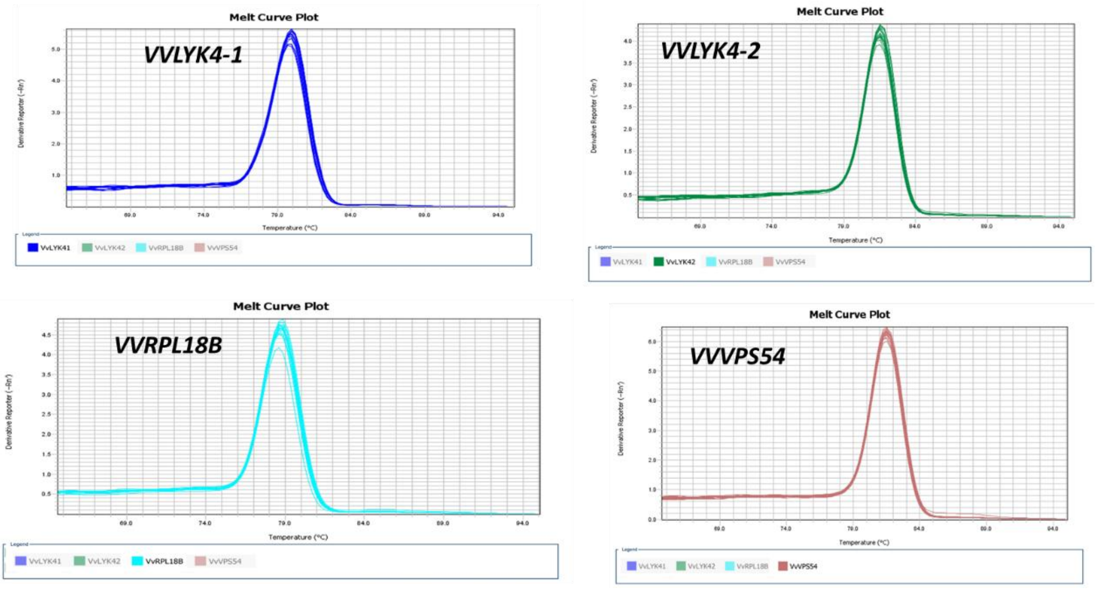

Melting curves for genes expression of Figure 2:

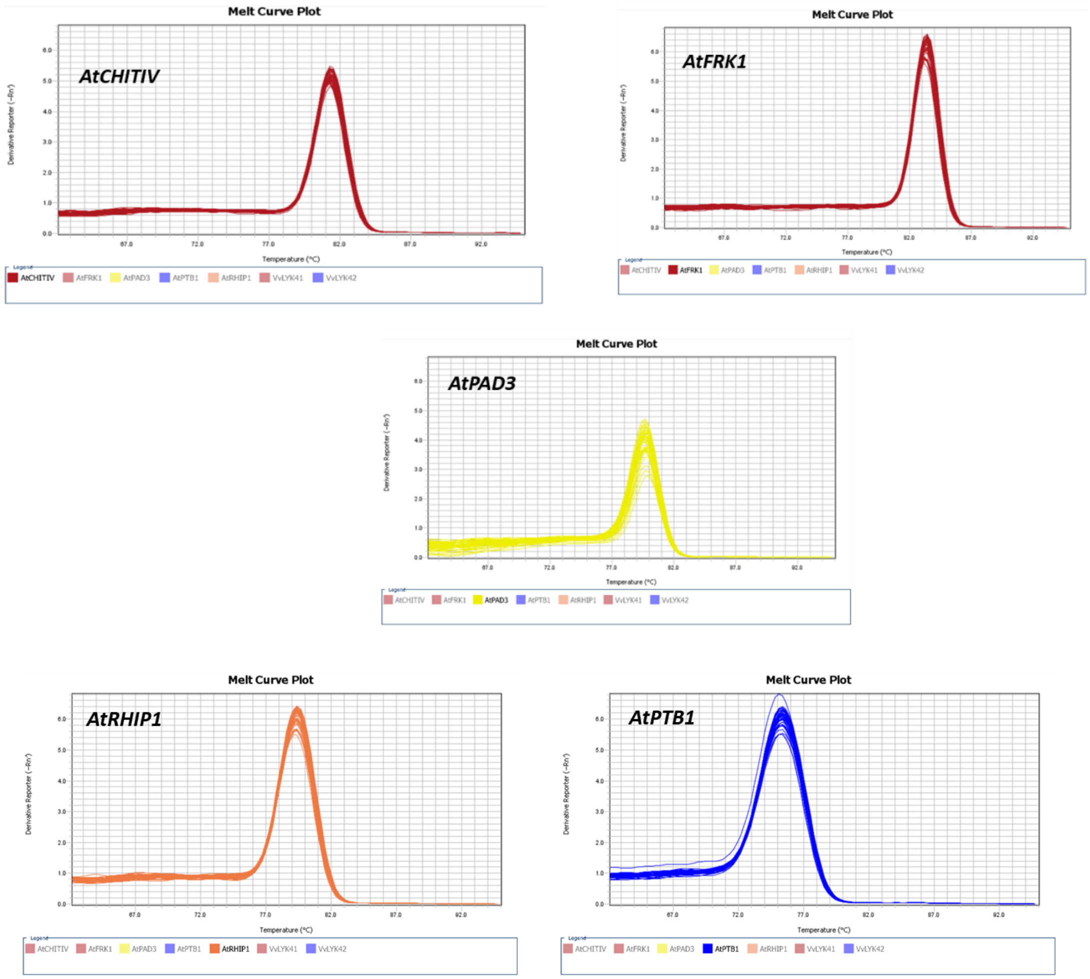

Melting curves for genes expression of Figure 5:

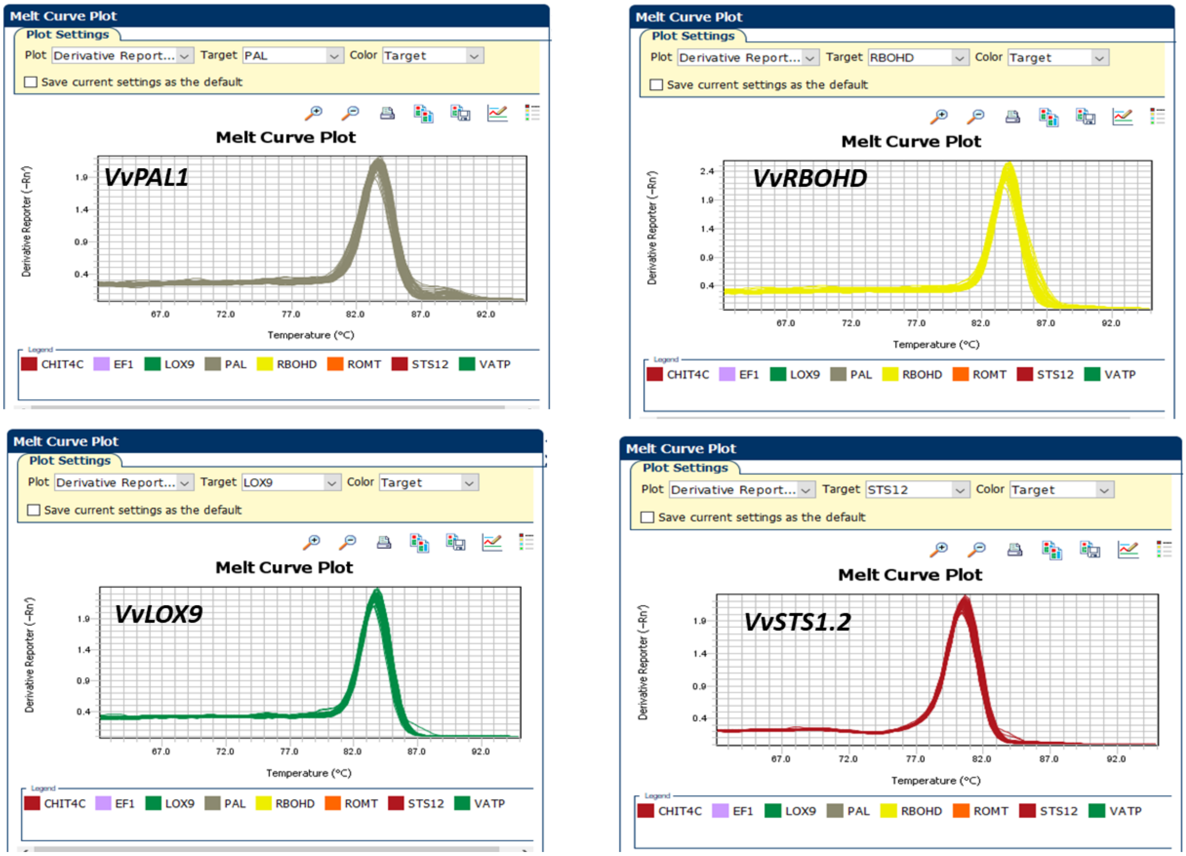

Figure S9. Melting curves of the different qPCR realized in this study.

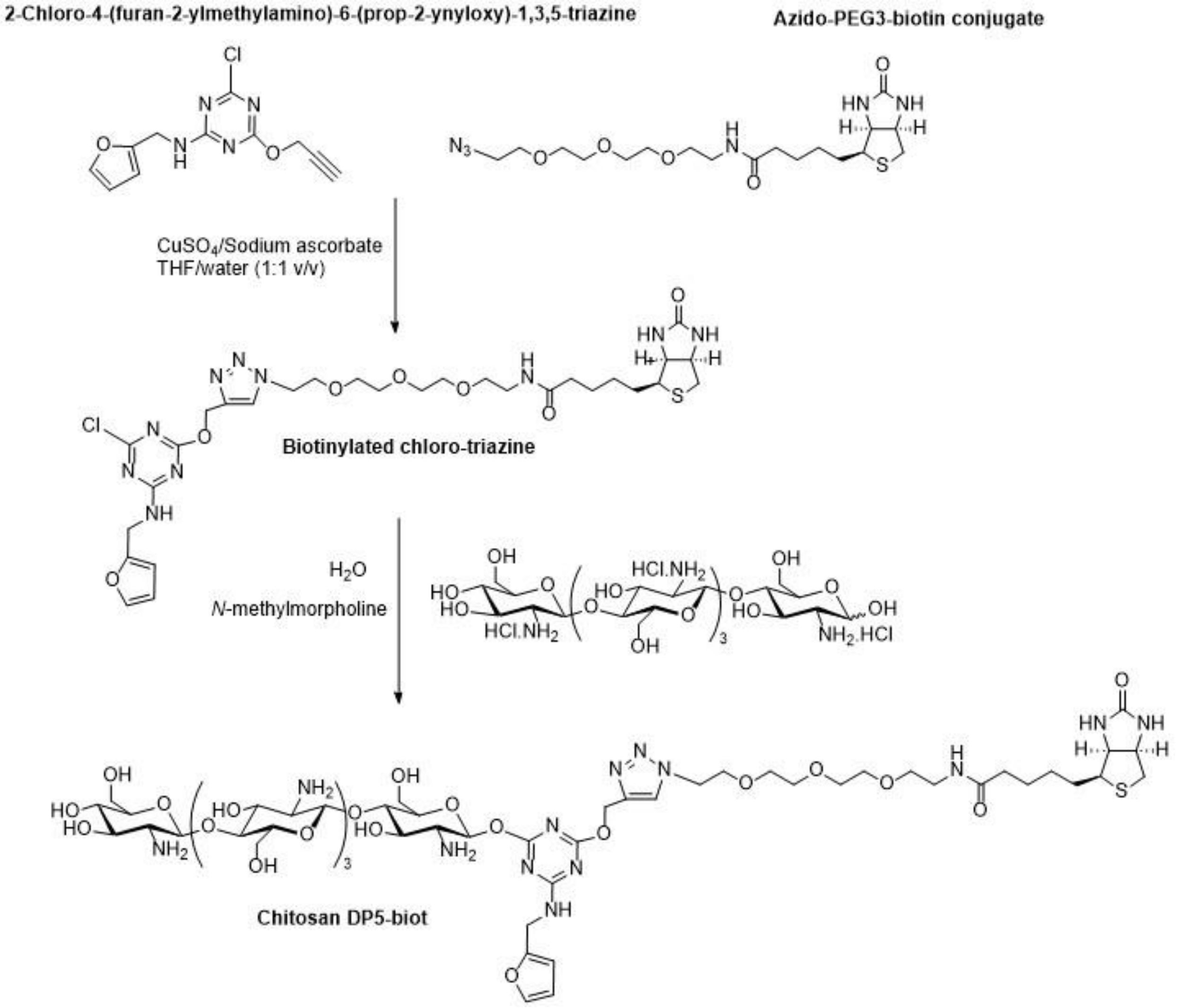

Figure S10. Synthesis of chitosan DP5-biot. Azido-PEG3-biotin conjugate (25 mg, 0.57 mmol) and 2-chloro-4-(furan-2-ylmethylamino)-6-(prop-2-ynyloxy)-1,3,5-triazine[1] (10 mg, 0.38 mmol) were solubilized in a THF/water 1:1 v/v solution (500 mL). Copper sulfate (57 mL of a 10 mg/mL solution in water, 0.23 mmol) and sodium ascorbate (45 mL of a 10 mg/mL solution in water, 0.23 mmol) were successively added and the reaction mixture was stirred overnight. The solution was concentrated and the residue was purified by flash chromatography on silica gel using a gradient of MeOH (5 to 10%) in dichloromethane as eluting system to provide pure biotinylated chloro-triazine (30 mg). ESI (positive mode) *m/z* : 731.41 [M+Na^+]

Chitopentaose pentahydrochloride (14 mg, 0.17 mmol) in water (1 mL) was added to a solution of biotinylated chloro-triazine (24 mg, 0.34 mmol). *N*-methylmorpholine (8 mL, 0.7 mmol) was added and the reaction mixture was stirred overnight. The solution was concentrated and the residue was purified by reversed-phase silica gel chromatography on a C18 SPE cartridge using a gradient of MeOH (0 to 40%) in water as eluting system. Pure (GlcNH2)5-triazine biotin (chitosan DP5-biot; 6.4 mg) was isolated and characterized by high-resolution mass spectrometry. HRMS (ESI) *m/z* calcd for $C_{59}H_{97}O_{28}N_{15}S$: 499.55222 $[M+3H]^{3+}$; found: 499.55264.

Chitopentaose pentahydrochloride was purchased from Seikagaku Co (Ref 400435, Lot No 9509120)

Azido-PEG3-biotin conjugate was purchased from Aldrich (Ref 762024).
